# Involvement of the Autophagy Protein Atg6 in Development and Virulence in the Gray Mold Fungus *Botrytis cinerea*

**DOI:** 10.3389/fmicb.2021.798363

**Published:** 2021-12-14

**Authors:** Na Liu, Shanyue Zhou, Baohua Li, Weichao Ren

**Affiliations:** ^1^College of Plant Health and Medicine, Qingdao Agricultural University, Qingdao, China; ^2^Engineering Research Center of Fruit and Vegetable Pest Precise Control of Qingdao, Qingdao, China

**Keywords:** *Botrytis cinerea*, autophagy, Atg6, development, pathogenicity

## Abstract

Gray mold caused by *Botrytis cinerea* is a devastating disease that leads to huge economic losses worldwide. Autophagy is an evolutionarily conserved process that maintains intracellular homeostasis through self-eating. In this study, we identified and characterized the biological function of the autophagy-related protein Atg6 in *B. cinerea*. Targeted deletion of the *BcATG6* gene showed block of autophagy and several phenotypic defects in aspects of mycelial growth, conidiation, sclerotial formation and virulence. All of the phenotypic defects were restored by targeted gene complementation. Taken together, these results suggest that BcAtg6 plays important roles in the regulation of various cellular processes in *B. cinerea*.

## Introduction

*Botrytis cinerea* (teleomorph *Botryotinia fuckeliana*) is a necrotrophic ascomycete fungus that causes serious pre- and postharvest crop losses worldwide to a large scope of plant species such as vegetables, fruits and ornamentals (Williamson et al., 2007; Dean et al., 2012). Due to the lack of resistant varieties, chemical control remains the most effective strategy for gray mold management, but many types of fungicides have failed by the genetic plasticity of *B. cinerea* (Williamson et al., 2007; Hu et al., 2016). Therefore, understanding the molecular mechanisms underlying development and virulence of *B. cinerea* will contribute to establish more effective disease control strategies. Moreover, *B. cinerea* has become a model fungus for molecular study of necrotrophic fungi (Tudzynski and Kokkelink, 2009).

Autophagy is a cellular degradation pathway for coping with nutritional stress and balancing energy sources in the process of cell differentiation (Glick et al., 2010). Autophagy also plays a housekeeping role in removing misfolded or aggregated proteins, clearing damaged organelles (such as endoplasmic reticulum, mitochondria and peroxisomes), and eliminating intracellular pathogens (Mizushima, 2007; Levine and Kroemer, 2008). To date, more than 40 autophagy-related proteins (ATGs) have been identified in yeast as regulating the initiation, nucleation, elongation and fusion of autophagy (Li and Vierstra, 2012; Klionsky et al., 2021). The initiation of autophagy is regulated by two protein complexes: the Unc-51-like autophagy-activating kinase (ULK) complex and the phosphoinositide 3-kinase (PI3K) complex III (Hurley and Young, 2017). The PI3K complex III catalyzes phosphatidylinositol 3-phosphate (PI3P) synthesis and recruits PI3P-binding proteins, especially the ATG18-ATG2 complex, for the initiation of autophagic membranes to autophagosome formation (Kihara et al., 2001; Suzuki et al., 2001). Yeast ATG6/vacuolar protein sorting 30 (VPS30), the ortholog of mammalian Beclin 1, is the key element of the PI3K complex along with VPS34, VPS15, and ATG14 (Kametaka et al., 1998).

The common role of Atg6 in the regulation of autophagy has been verified in yeast, plants and animals, and the yeast Atg6 is also required for the sorting of vacuolar hydrolases (Furuya et al., 2010). Arabidopsis Atg6 regulates normal growth, pollen germination and responses to biotic/abiotic stresses (Fujiki et al., 2007; Qin et al., 2007). Beclin 1 functions as a tumor suppressor in mammals (Qu et al., 2003; Yue et al., 2003). For pathogenic fungi, Atg6 plays an important role in vegetative differentiation and pathogenesis in the rice blast fungus *Magnaporthe oryzae* and the Fusarium head blight fungus *Fusarium graminearum* (Kershaw and Talbot, 2009; Lv et al., 2017).

Despite the growing interest in Atg6/Beclin 1, the knowledge about Atg6 in the model fungus *B. cinerea* remains unknown. In this study, we identified and characterized BcAtg6 in *B. cinerea*, and determined its role in autophagy, fungal development and pathogenicity.

## Materials and Methods

### Strains and Culture Conditions

The *B. cinerea* wild-type strain B05.10 was used as parental strain for genetic modifications. B05.10 and the derivative strains were cultured on potato dextrose agar (PDA), minimal medium (MM), and complete medium (CM), as described previously (Ren et al., 2018a). PDA and sterilized potato fragments were used to determine conidiation. PDA and MM media were used to determine sclerotial formation. MM-N [MM without (NH_4_)_2_SO_4_] was used for induction of autophagy.

### Gene Deletion and Complementation

To replace *BcATG6* in the wild-type strain B05.10, 1,372-bp upstream and 1,281-bp downstream flanking sequences of *BcATG6* were amplifies by PCR from the genomic DNA of B05.10. The resulting amplicons were fused with *HPH* (hygromycin resistance gene) by using double-joint PCR (Yu et al., 2004). Protoplast preparation and transformation were performed as the previously described method (Gronover et al., 2001). The resulting hygromycin-resistant transformants were preliminarily screened by PCR with primers (Supplementary Table 1), and further confirmed by Southern blotting analysis. The upstream fragment of *BcATG6* was used as a probe and labeled with digoxigenin (DIG) using the High Prime DNA Labeling and Detection Starter Kit II, according to the protocol of the manufacturer (Roche Diagnostics, Mannheim, Germany). The genomic DNA was digested with BamH1 endonuclease. For complementation assays, BcAtg6-GFP cassette was generated as described previously (Ren et al., 2018b). Briefly, the entire ORF (open reading frame) of *BcATG6* (without stop codon) was amplified and cloned into pNAN-OGG vector containing the GFP allele and the nourseothricin resistance gene. The resulting construct was confirmed by sequencing and transformed into the *BcATG6* deletion mutant.

### Western Blotting Assay

The total proteins of the GFP-BcAtg8 fusion protein expressing strains under nutrient-rich and nitrogen starvation conditions were extracted as previously described (Gu et al., 2015) and equal amounts of proteins were loaded into each lane of a 10% sodium dodecyl sulfate-polyacrylamide gel. After electrophoresis, proteins were transferred onto Immobilon-P transfer membrane (Millipore, Billerica, MA, United States) with a Bio-Rad electroblotting apparatus. The anti-GFP antibody (Abcam, Cambridge, Cat#Ab32146) and anti-GAPDH antibody (Hangzhou Huaan Biotechnology Co., Ltd., Hangzhou, China, Cat #EM1101) were used at 1: 5,000–1: 10,000 dilution for immunoblot analyses. All experiments were performed at least three times.

### Pathogenicity Assay

Infection tests were performed on strawberry fruits and cucumber leaves. Briefly, the tested plant tissues were point-inoculated with 5-mm diameter mycelial plugs of 3-day-old cultures. Before inoculation, the cuticle of hosts was wounded with a sterilized needle tip to facilitate penetration of the fungus into plant tissues. Additionally, water agar plugs without fungal mycelia were used as negative controls (mock). The inoculated samples were placed in a high relative humidity condition (about 95%) at 25°C with 16 h of daylight. These experiments were repeated three times and each time with at least ten samples.

## Results

### Identification of BcAtg6 in *Botrytis cinerea*

**FIGURE 1:**
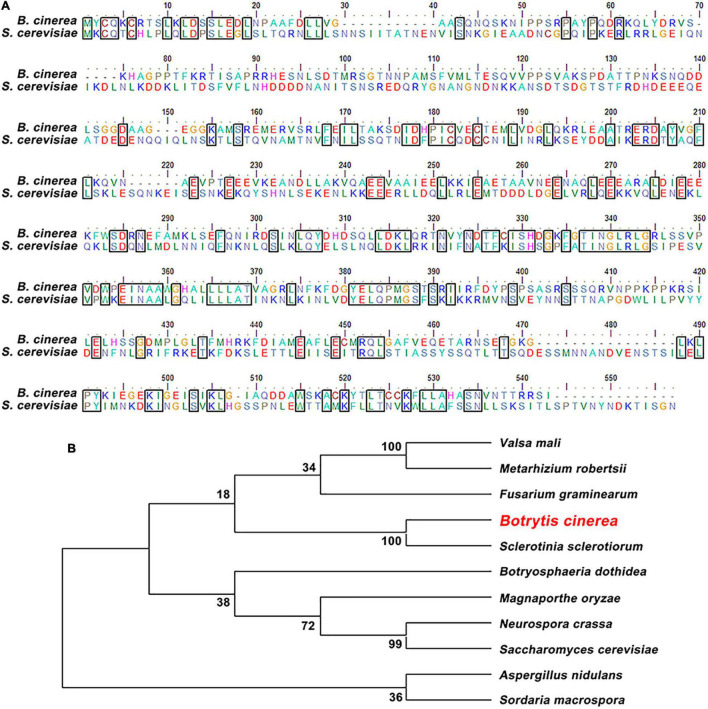
Sequence alignment and phylogenetic analysis of BcAtg6. **(A)** Protein sequence alignment of Atg6 orthologs from *Botrytis cinerea* and *Saccharomyces cerevisiae*. The identical amino acids are in a same box. **(B)** Phylogenetic analysis of BcAtg6 and its orthologs, including *Valsa mali*, *Metarhizium robertsii*, *Fusarium graminearum*, *Sclerotinia sclerotiorum*, *Botryosphaeria dothidea*, *Magnaporthe oryzae*, *Neurospora crassa*, *Aspergillus nidulans*, *Sordaria macrospora*, and *Saccharomyces cerevisiae*. The phylogenetic tree was constructed by MEGA7 software using neighbor-joining method.

The BcAtg6 protein coding gene *BcATG6* (BCIN_05g05500) was retrieved based on BLASTP search of the genome database of *B. cinerea*^1^ with the *Saccharomyces cerevisiae* Atg6 protein as a query. *BcATG6* was predicted to encode a 501 amino-acid protein, which shares 32% identity with *S. cerevisiae* Atg6 (Figure 1A). Phylogenetic analysis of BcAtg6 with other Atg6 homologs from different fungal species revealed that BcAtg6 is evolved conservatively in fungi kingdom (Figure 1B).

**FIGURE 2:**
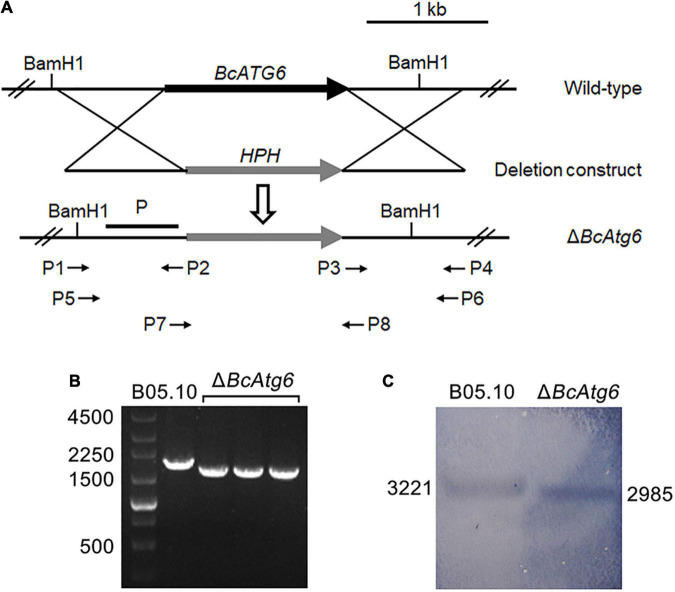
Target gene deletion of *BcATG6*. **(A)** Schematic diagram of the *BcATG6* homologous replacement strategy. **(B)** PCR-based screening of the putative *BcATG6* deletion mutants using P7/P8 primer. **(C)** Southern blotting analysis of the *BcATG6* deletion mutants using a *BcATG6* upstream fragment as probe and the genomic DNA was digested with BamH1 endonuclease.

To investigate the role of *BcATG6* in *B. cinerea*, the targeted gene deletion mutants of *BcATG6* were generated using a homologous recombination strategy (Figure 2A). The putative *BcATG6* deletion mutants were selected from the hygromycin-resistant transformants by PCR analysis (Figure 2B). Southern blotting further confirmed the right recombination event of Δ*BcAtg6* occurred at the *BcATG6* locus (Figure 2C).

### BcAtg6 Is Required for Autophagy

**FIGURE 3:**
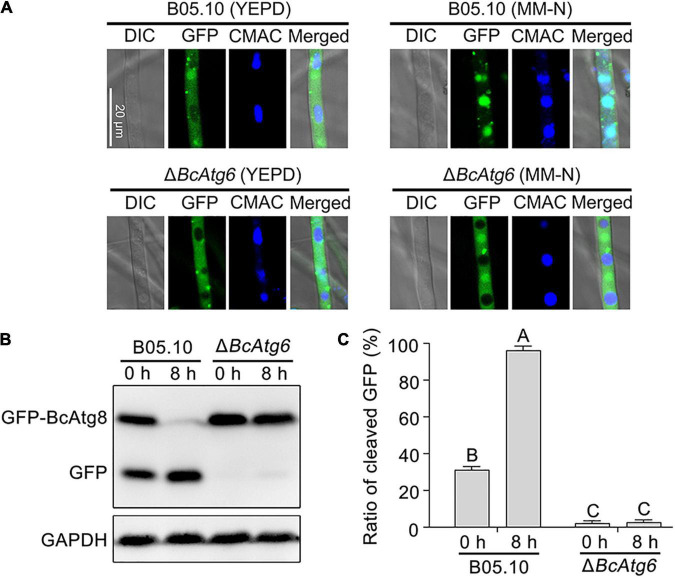
BcAtg6 is essential for autophagy. **(A)** GFP-BcAtg8 localization in mycelia of the wild-type strain B05.10 and *BcATG6* deletion mutant Δ*BcAtg6* that treated under nutrient-rich and nitrogen starvation conditions. The vacuoles were stained with CMAC (7-amino-4-chloromethylcoumarin). **(B)** GFP-BcAtg8 proteolysis of each strain (starvation for 0 and 8 h) was analyzed by Western blotting using an anti-GFP antibody. GAPDH was used as an internal reference. **(C)** The percentage of GFP on the total of GFP and GFP-BcAtg8. Error bars indicate standard deviation from three independent experiments. Values on the bars followed by the same letter are not significantly different at *P* = 0.05.

GFP-BcAtg8 is a useable marker to monitor autophagy in *B. cinerea* (Ren et al., 2018a). To determine the role of BcAtg6 in autophagy, the proteolysis of GFP-BcAtg8 was analyzed. Under nutrient-rich conditions, the GFP-BcAtg8 fusion protein and free GFP protein were detected by anti-GFP western blotting in the wild-type strain B05.10, and nitrogen starvation promoted the GFP-BcAtg8 proteolysis. However, nitrogen starvation had no effect on the protein contents of GFP-BcAtg8 and GFP in the *BcATG6* deletion mutant Δ*BcAtg6* (Figure 3). These results indicate that BcAtg6 plays an important role in the regulation of autophagy in *B. cinerea*.

### BcAtg6 Is Involved in Vegetative Growth

**FIGURE 4:**
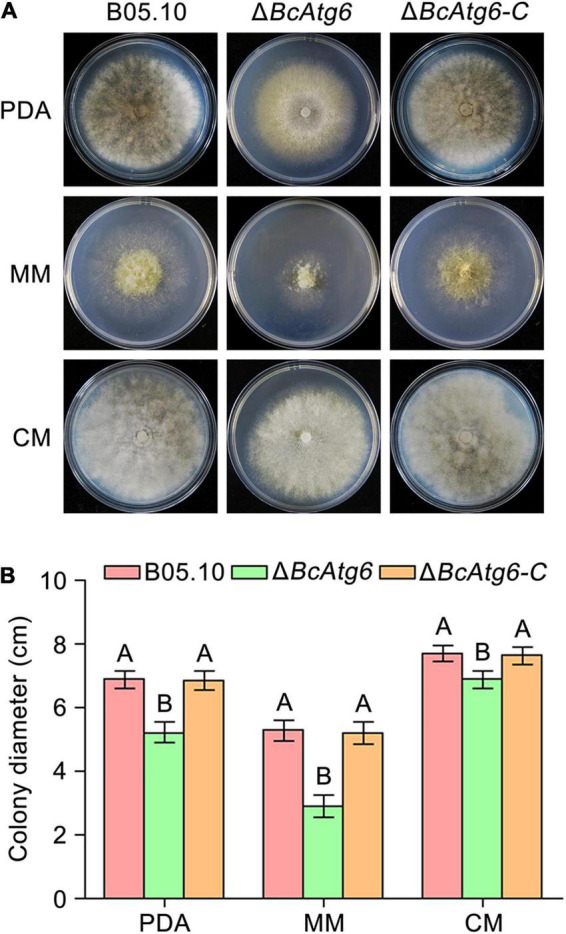
BcAtg6 regulates vegetative growth. **(A)** Colony morphology of the wild-type strain B05.10, *BcATG6* deletion mutant Δ*BcAtg6* and complemented strain Δ*BcAtg6-C* growing on PDA, MM, and CM media at 25°C for 3 days. **(B)** The mycelial growth rates of each strain on various media. Error bars indicate standard deviation from three independent experiments. Values on the bars followed by the same letter are not significantly different at *P* = 0.05.

To determine the role of BcAtg6 in growth, the wild-type strain B05.10, *BcATG6* deletion mutant Δ*BcAtg6* and complemented strain Δ*BcAtg6-C* were cultured on PDA, MM and CM media. After incubation at 25°C for 3 days, Δ*BcAtg6* exhibited a distinct colony morphology with fewer aerial mycelia (Figure 4A), and a decreased colony radial growth rate compared with B05.10 and Δ*BcAtg6-C* (Figure 4B). These results indicate that BcAtg6 is important for vegetative growth in *B. cinerea*.

### BcAtg6 Is Involved in Conidiation

**FIGURE 5:**
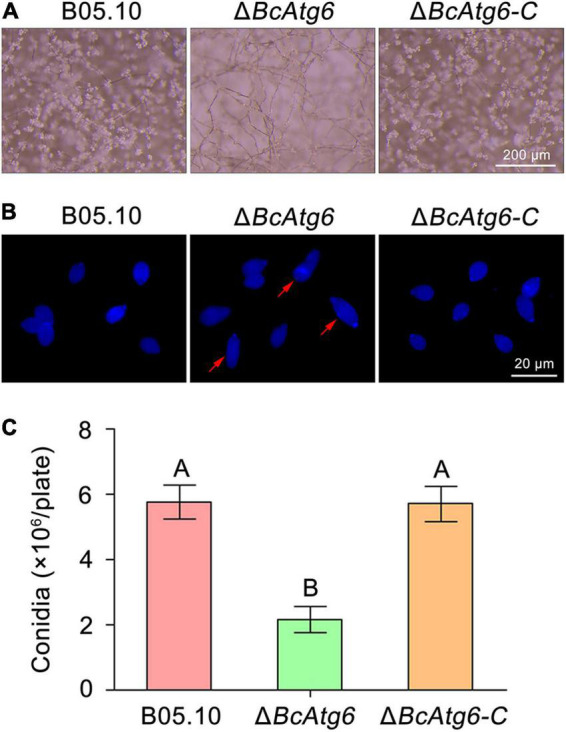
BcAtg6 regulates conidiation. **(A)** Conidia on conidiophores of the wild-type strain B05.10, *BcATG6* deletion mutant Δ*BcAtg6* and complemented strain Δ*BcAtg6-C* were observed after 7 days of incubation on sterilized potato fragments at 25°C with a 12-h photophase. **(B)** Conidial morphology of each strain. Red arrows indicate abnormal conidia. **(C)** Quantification of the conidia produced by each strain on sterilized potato fragments. Error bars indicate standard deviation from three independent experiments. Values on the bars followed by the same letter are not significantly different at *P* = 0.05.

To determine the role of BcAtg6 in conidiation, the wild-type strain B05.10, *BcATG6* deletion mutant Δ*BcAtg6* and complemented strain Δ*BcAtg6-C* were cultured on sterilized potato chips. After incubation at 20°C under white light for 10 days, Both B05.10 and Δ*BcAtg6-C* formed a dense layer of mycelia covered with lots of conidia, while Δ*BcAtg6* failed to form a conidial layer (Figure 5A). The conidiation of Δ*BcAtg6* was significantly reduced compared with that of B05.10 and Δ*BcAtg6-C* (Figure 5B). In addition, Δ*BcAtg6* produced some deformed conidia (Figure 5C). These results indicate that BcAtg6 is important for conidiation in *B. cinerea*.

### BcAtg6 Is Involved in Sclerotial Formation

**FIGURE 6:**
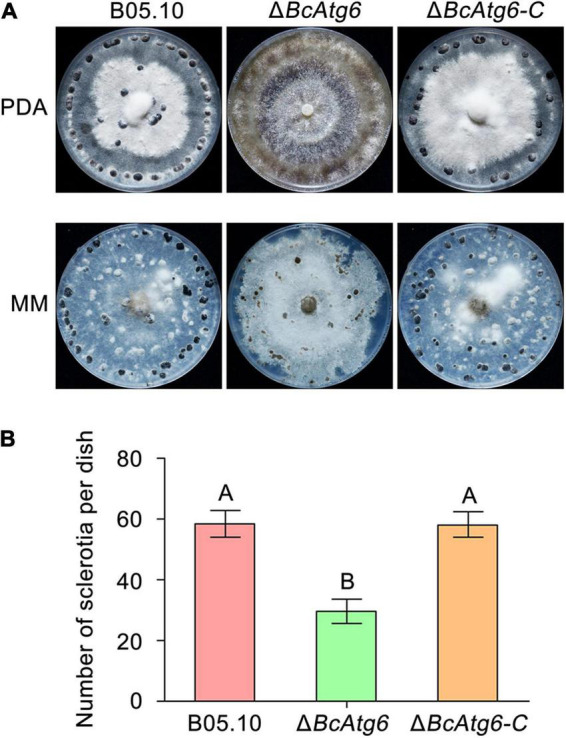
BcAtg6 regulates sclerotial formation. **(A)** Sclerotial formation of the wild-type strain B05.10, *BcATG6* deletion mutant Δ*BcAtg6* and complemented strain Δ*BcAtg6-C* after 3 weeks of incubation on PDA and MM media at 10°C in the dark. **(B)** The number of sclerotia produced by each strain on MM media. Error bars indicate standard deviation from three independent experiments. Values on the bars followed by the same letter are not significantly different at *P* = 0.05.

Sclerotium is a structure that allows the fungus to survive under unfavorable conditions (such as over the winter) (Williamson et al., 2007). To determine the role of BcAtg6 in sclerotial formation, the wild-type strain B05.10, *BcATG6* deletion mutant Δ*BcAtg6* and complemented strain Δ*BcAtg6-C* were culture on PDA and MM media. After incubation at 10°C in the dark for 3 weeks, B05.10 and Δ*BcAtg6-C* were able to form sclerotia on both PDA and MM media, while Δ*BcAtg6* only formed sclerotia on MM medium (Figure 6A), and the amount of sclerotia produced by Δ*BcAtg6* on MM medium was significantly reduced compared with that of B05.10 and Δ*BcAtg6-C* (Figure 6B). These results indicate that BcAtg6 is important for sclerotial formation in *B. cinerea*.

### BcAtg6 Is Required for Virulence

**FIGURE 7:**
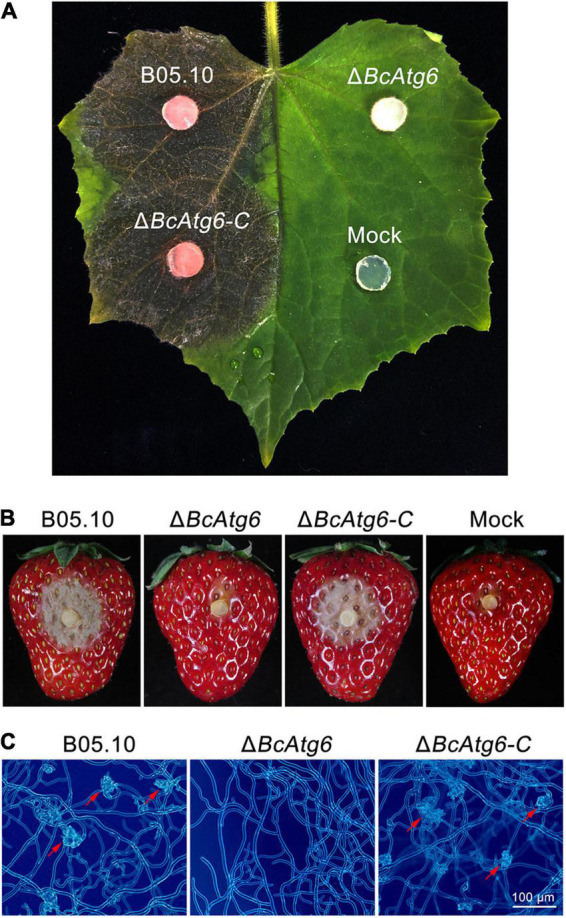
BcAtg6 is required for virulence. **(A)** Cucumber leaves were inoculated with the wild-type strain B05.10, *BcATG6* deletion mutant Δ*BcAtg6* and complemented strain Δ*BcAtg6-C* for 3 days. **(B)** Strawberry fruits were inoculated with each strain for 3 days. **(C)** The infection structures formed by each strain in early infection stage. Red arrows indicate infection structures.

Autophagy plays an important role in virulence of pathogenic fungi (Liu et al., 2016). To determine the role of BcAtg6 in virulence of *B. cinerea*, cucumber leaves and strawberry fruits were inoculated with the wild-type strain B05.10, *BcATG6* deletion mutant Δ*BcAtg6* and complemented strain Δ*BcAtg6-C*. After 3 days post-inoculation (dpi), B05.10 and Δ*BcAtg6-C* caused typical symptoms on both cucumber leaves and strawberry fruits, while Δ*BcAtg6* failed to infect those two host tissues (Figures 7A,B). In addition, Δ*BcAtg6* lost ability to form infection structures at the initial stage of infection (Figure 7C). These results indicate that BcAtg6 is essential for virulence in *B. cinerea*.

## Discussion

The most primordial function of autophagy is to recycle proteins and organelles within cells as an adaptation to nutrient deprivation (Klionsky, 2007). With the deepening of research, more and more autophagy functions have been discovered in eukaryotes including plant pathogenic fungi (Yang and Klionsky, 2009). In this study, we focused on BcAtg6, a hitherto uncharacterized protein in the gray mold fungus *B. cinerea*. Reverse genetic analysis showed that BcAtg6 is essential for autophagy and plays important roles in mycelial growth, conidiation, sclerotial formation and virulence which are consistent with the previous reports of the role of autophagy in *B. cinerea* (Liu et al., 2019).

As a core component of the class III phosphatidylinositol 3-kinase (PI3K-III) complex, Atg6 is essential for autophagy and the phosphatidylinositol-3-phosphate (PI3P) signaling pathways (Yue et al., 2015). Previously, fungal Atg6 orthologs had been identified and characterized in *M. oryzae* and *F. graminearum*, and their necessity in autophagy had been demonstrated (Kershaw and Talbot, 2009; Lv et al., 2017). In this study, loss of BcAtg6 blocked autophagy in *B. cinerea* suggesting that the function of BcAtg6 in autophagy is evolutionarily conserved.

Endogenous recycling of the cellular constituents by autophagy is crucial for the normal life of fungi and autophagy has been reported to be involved in the regulation of mycelial growth and development in some filamentous fungi (Pollack et al., 2009; Khan et al., 2012). In this study, the Δ*BcAtg6* mutant exhibited severe defects in mycelial growth and conidiation, which is consistent with the Δ*Atg6* mutants in *M. oryzae* and *F. graminearum* (Kershaw and Talbot, 2009; Lv et al., 2017). It is worth noting that the Δ*BcAtg6* mutant has the ability to produce sclerotia only on a medium of specific nutrients. These results indicate that autophagy-mediated intracellular recycling plays an important role in supporting the normal vegetative growth and differentiation in *B. cinerea*.

Autophagy plays an important role in morphogenesis of pathogenic fungi during the initial infection stage (Pollack et al., 2009). Previous studies have shown that the main reason for reduced virulence of the autophagy blocked mutants in *M. oryzae* and *M. robertsii* was the appressorium formation defects in the early infection stage (Liu et al., 2007; Duan et al., 2013). In this study, the Δ*BcAtg6* mutant cannot form infection structures in the early infection stage and therefore lost pathogenicity. These results indicate that the infection structure morphogenesis mediated by autophagy is required for virulence in *B. cinerea*.

## Conclusion

In conclusion, our results indicate that BcAtg6 is involved in the regulation of fungal development and virulence in *B. cinerea*.

## Data Availability Statement

The original contributions presented in the study are included in the article/Supplementary Material, further inquiries can be directed to the corresponding author/s.

## Author Contributions

BL and WR conceived and designed the study. NL and SZ performed the experiments. NL and WR analyzed the data and wrote the manuscript. All authors read and approved the manuscript.

## Conflict of Interest

The authors declare that the research was conducted in the absence of any commercial or financial relationships that could be construed as a potential conflict of interest.

## Publisher’s Note

All claims expressed in this article are solely those of the authors and do not necessarily represent those of their affiliated organizations, or those of the publisher, the editors and the reviewers. Any product that may be evaluated in this article, or claim that may be made by its manufacturer, is not guaranteed or endorsed by the publisher.

## Footnotes

1
https://fungi.ensembl.org/Botrytis_cinerea/Info/Index
