# Supplementary material for: Involvement of the Autophagy Protein Atg6 in Development and Virulence in the Gray Mold Fungus Botrytis cinerea
Source: Front Microbiol. 2021 Dec 14;12:798363. doi: 10.3389/fmicb.2021.798363 (PMC8712751; doi:10.3389/fmicb.2021.798363)
Supplement: Supplementary file 1 [file Table_1.DOC]

**Table S1**. Oligonucleotide primers used in this study.

| Primer | Sequence (5’-3’) | Relevant characteristics |
| --- | --- | --- |
| P1 | GCTCAGTTTCTGGCTCTG | PCR primers to amplify *BcATG6* upstream fragment for construction of the gene deletion vector |
| P2 | CAAAATAGGCATTGATGTGTTGACCTCCTTGTGGATGTTTGGCTGG |
|  |  |  |
| P3 | CTCGTCCGAGGGCAAAGGAATAGAGTAGGATCATTGATGCACAGGG | PCR primers to amplify *BcATG6* downstream fragment for construction of the gene deletion vector  PCR primers to amplify the hygromycin B B (*HPH*) gene  PCR primers to amplify the deletion vector of *BcATG6* with the double-joint PCR products as template  PCR primers to identificate the *BcATG6* deletion mutant  PCR primers to amplify the probe fragment for *BcATG6* Southern blotting  PCR primers to amplify the full length coding region of *BcATG6* for construction of the BcAtg6-GFP vector  PCR primers for identification of the in-frame BcAtg6-GFP fusion vector |
| P4  HPH-F  HPH-R  P5  P6  P7  P8  P9  P10  P11  P12  P13  P14 | CTAACTAACTAGGTAGATG  GGAGGTCAACACATCAATGC  CTACTCTATTCCTTTGCCCT  CGACTCATCGTAGCCACG  CCATCATTACCTCCAGCTC  GTGAGTGAATCTACAACG  CAGTCCAAGTGCCAGGAAC  CCTCCACATTGCTATATTCG  GTCCATCAAAGGTCTAAGAG  CCATCACATCACAATCGATCCAA  CCATGTATTGTCAAAAGTGTCG  TACTTACCTCACCCTTGGAAACC  ATTATACTCCTCCTCGTAGTAT  GTTGAGCAGGAAACTGCCAG  CCTCTGGCATTGCAGACTTG |
